# Treatment of Extensively Comminuted Mandibular Fracture with the Aid of a Condylar Positioning Device

**DOI:** 10.1155/2017/2732907

**Published:** 2017-12-17

**Authors:** Abd Jabar Nazimi, Tengku Ezulia, Jothi Raamahlingam Rajaran, Siti Salmiah Mohd Yunus, Syed Nabil

**Affiliations:** ^1^Department of Oral and Maxillofacial Surgery, Universiti Kebangsaan Malaysia Medical Centre, Cheras, 56000 Kuala Lumpur, Malaysia; ^2^Department of Otorhinolaryngology, University of Malaya Medical Centre, 59100 Kuala Lumpur, Malaysia

## Abstract

We describe a case of extensively comminuted mandibular fracture that extends bilaterally to the angle of mandible successfully treated with the use of condylar positioning device (CPD). This simple, yet effective, technique that almost exclusively described in orthognathic surgery is useful when advance surgical techniques such as pre- or intraoperative landmark identification may not be readily available. CPD technique optimizes the manual manipulations of the comminuted distal segments during fracture reduction and internal fixation. At the same time, it allows greater control of the proximal segments to avoid further surgical complication.

## 1. Introduction

Mandibular fractures are a frequent injury sustained when there is trauma to the face and jaw. This could lead to functional and aesthetic problems. Oliveira and coworkers [1] reiterated that the high incidence of mandibular fracture was related to its anatomy and characteristics. The term “comminuted fracture” is used when there are multiple fracture lines that result in many small pieces of bones within the same areas [2]. Extensive communited fracture is regarded when the fracture involves multiple sites that exceed the region or when the neighbouring region is also involved [3]. Complex, extensively comminuted, and pulverized fractures are commonly seen in a high-velocity and energy impact as in a fall from height. The decision for the appropriate treatment can be complicated when there are no reference points for the reduction to be performed apart from the dental occlusion. However, it is often seen in an extensively comminuted mandibular fracture that the occlusion cannot be exactly characterized although maxilla-mandibular fixation (MMF) has been recognized as important component of therapy [2]. It is also somewhat critical as traditionally, one have to avoid unnecessary or excessive periosteal stripping that may devascularize the small bone segment and at the same time to completely immobilize the major fracture segments [4]. To obtain surgical goals, it is also necessary for the surgeon to attain good accessibility of the fracture sites and to ensure that both of the proximal segments are in the correct position. Moreover, exact algorithm for fracture reduction and fixation in this surgical situation has never been established except for standard principles such as the use of load bearing, locking plates, and small plates in simplifying the fractures.

The purpose of this paper is to describe the usefulness of the condylar positioning device (CPD) that was almost exclusively described in orthognathic surgery in stabilizing the proximal segments in an extensively comminuted mandible fracture reduction and fixation.

## 2. Case Report

A 32-year-old lady fell from height and sustained deep laceration wound on the chin compounded with extensively comminuted mandible fracture. The fracture extends to the mandibular angle bilaterally causing severely deranged occlusion. She also sustained a medially displaced left condylar head fracture and an undisplaced left Le Fort II fracture.

An open reduction and internal fixation (ORIF) was planned for her comminuted mandible fracture by using a prebent reconstruction plate through the existing wound. ORIF for both left Le Fort II and condylar fracture was also planned. The aim was first to establish the proper vertical dimension and posterior mandibular height prior to the fixation of the comminuted mandible. However, adding to the difficulties in the execution of the surgical plan, our patient refused for both ORIF of the maxilla and condylar fracture, as it requires additional transcutaneous surgical access. She previously experienced complicated emergency procedure for open fracture of the left supracondylar femur and requested fixation only for her mandible fracture. We therefore have to revise the protocol required for her surgery and decided to use the CPD as a measure to avoid rotational movements of proximal segments of the mandible, as these were the only surgical reference left.

**Figure 1:**
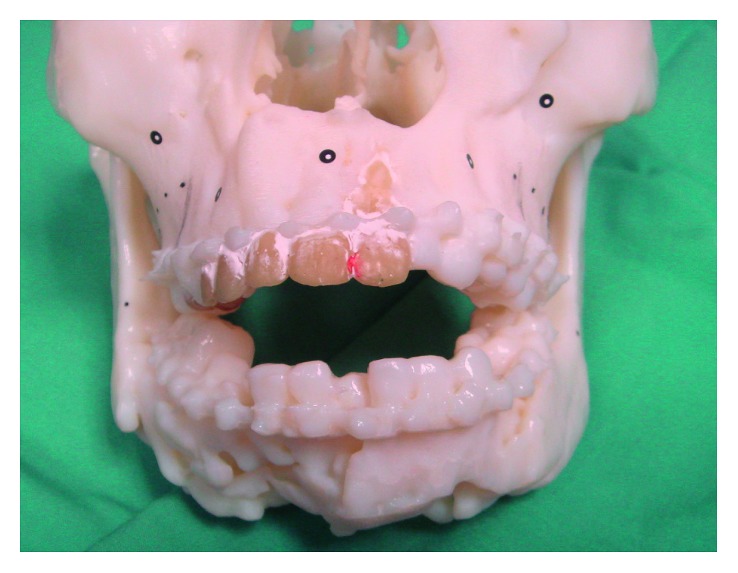
Acrylonitrile butadiene styrene (ABS) model showing comminuted mandible fracture with severe anterior open bite.

To begin with, we applied an additive manufacturing (AM) concept to produce an acrylonitrile butadiene styrene (ABS) three-dimensional (3D) life-size surgical model by using her DICOM computed tomography (CT) data (Figure 1). From the clinical assessment and having the printed model in hand, we could confirm that we were unable to find any intact anatomical references needed for fracture reduction and fixation nor that we possess the preoperative landmark identification or an intraoperative tool by using a navigation system or imaging to check for the exact and adequacy of bony reduction and position.

Dental occlusion as the most important reference was also lost. Clinically, our patient had 30 mm anterior open bite (AOB) with only the last remaining molar in the occlusion. Loss of left posterior facial height was also observed secondary to condylar neck fracture. Hence, we decided to use the only remaining occlusion on the molar teeth and construct the CPD to make sure that these two remaining references will not change during the surgical procedure that could risk the surgical outcome. Without the use of any feasible technique to gain control of the proximal segment, there is a high risk of malocclusion and proximal fragment positional change. This could lead to more surgical complications such as temporomandibular joint (TMJ) pain, TMJ compression, condylar resorption, and the need for reoperative procedure.

**Figure 2:**
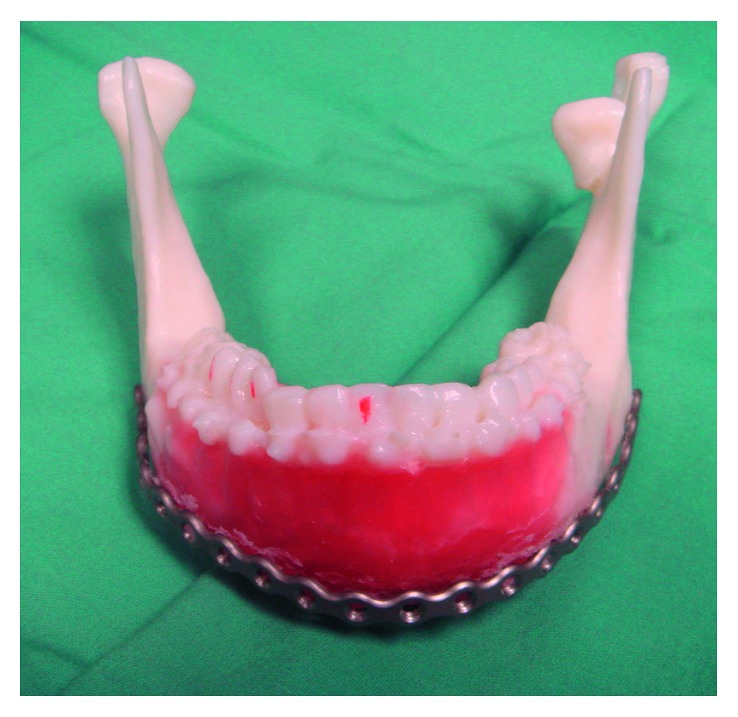
Fracture reduction performed following angle mandible osteotomy and then waxing-up to allow adaptation of the reconstruction plate. Note that there is loss of left posterior facial height secondary to condylar neck fracture.

By using the ABS model, we adapted the Luhr [5] technique of CPD bilaterally by using 2.0 rigid fixation plate in the centric condylar position. We then simulated the osteotomy located at the most posterior aspect of the mandibular fracture with both condyles left undisturbed within the glenoid fossae. Next, fracture reduction and correction of occlusion were carried out, followed by waxing-up of the multiple defect areas to allow good bending and adaptation of the reconstruction plate (Figure 2).

**Figure 3:**
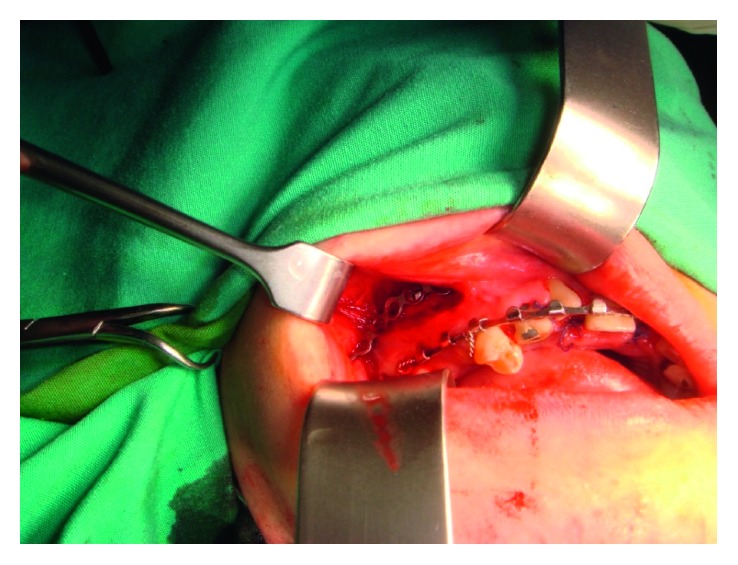
Intraoperative condylar positioning device on right maxilla. This procedure was completed bilaterally prior to the surgery on the mandible in stabilizing the proximal segments bilaterally.

In the operating theatre, general anaesthesia was delivered via fibreoptic nasal intubation to minimize manipulation of the mandible. Once under general anaesthesia, vestibular incision was first made on the posterior maxilla and mandible bilaterally for CPD adaptation and fixation. This was the first step undertaken to prevent any unnecessary change in the condylar position before carrying out further surgical procedures (Figure 3).

**Figure 4:**
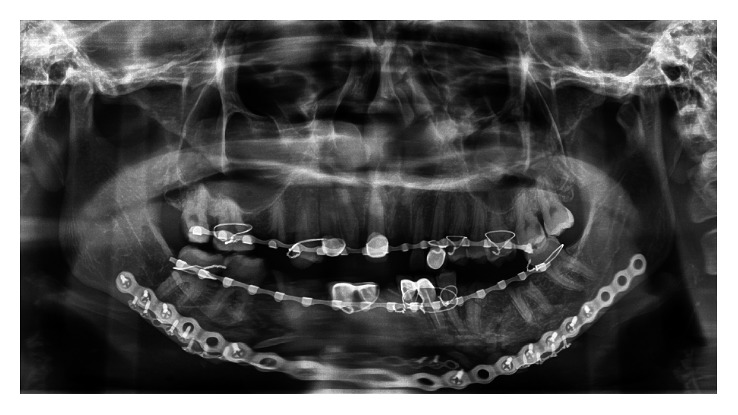
Postoperative panoramic showing good fracture reduction and occlusion achieved.

For access to fracture sites and for reduction and internal fixation, the existing chin laceration wound was used and extended posteriorly reaching the now-stabilized proximal segments. This followed by manipulation and reduction of the comminuted fragments. Fixations were completed with the prebent locking mandibular reconstruction plate. Small fragments that were amenable for fixation were simplified using microplates and screws. The CPD was temporarily relieved, allowing us to examine the final occlusion and jaw motions following completion of fixations. We found that the CPD encouraged appreciable, undisturbed condylar position as the desired occlusion, jaw opening, and excursion were achieved. The CPD was removed, and the incision closed in the usual manner. Postoperative follow-up and orthopantomogram showed good healing with restoration of her preinjury states of occlusion (Figure 4). Clinically, good mouth opening was achieved with no deviation during mouth opening.

## 3. Discussion

Treatment of comminuted mandibular fractures remains a challenge even for experienced surgeons. Difficulties exist in establishing accurate reduction and fixation of the fragments, especially when there is complete loss of anatomic references or occlusal relationship [6].

In preserving the vascularity to the comminuted fragments and preventing secondary infections, closed reduction has long been considered as the treatment of choice. However, recent reports insisted that open reduction and internal fixation (ORIF) is a better treatment option with less complication rates [7, 8]. The advancement in surgical method and options for more robust and reliable internal fixation method has favoured for ORIF in the management of a comminuted mandibular fracture. It was also suggested that closed reduction or conservative treatment is a better choice only when there is minimally displaced comminuted fractures [9].

In addition, when the fracture involves mandibular condyles, early mobilization of the jaw and functional rehabilitation are essential due to risk of developing functional disturbances secondary to the adhesion, fibrosis, and ankylosis [10]. These complications were in general far more complex to be treated if occurred.

The application of CPD or also known as proximal segment positioning plate technique has been widely discussed in orthognathic surgery [11–14]. However, considering its importance to avoid complications associated with malposition of the condylar head when anterior or distal segment of the mandible is being manipulated, we found no previous publications designated the use of such a technique in severe trauma cases. The other technique that was previously described, often used in mandibulectomy oncological surgery, is to have the plate transversing the anterior segment of the mandible. This, however, may be not suitable in a severely comminuted mandible fracture as the presence of another large plate or fixation device within the same area could hinder good access for fracture manipulation, reduction, and internal fixation. In addition, the placement of CPD in the maxilla and posterior mandible could overcome the situation where the guiding plate may preclude the areas intended for the placement of reconstruction plate and screws. This was imminent in the comminuted fracture as the amount of bony stock available for fixation could be very limited.

Overall, this case was successfully treated by using CPD technique taking into consideration various important aspects in the treatment of mandibular fracture. This includes early mobilization in concomitant condylar fracture, restoration of the occlusion, and reestablishing the posterior ramus height. Only a short period of maxillomandibular fixation was required, and early TMJ rehabilitation is possible in this elaborated case.

CPD technique allows precise intraoperative control of the proximal segments. Without good control, it could lead to many debilitating mandibular fracture complications such as loss of the gonial angle, loss of posterior facial height, condylar sag, pain, dysfunction of the temporomandibular joint, and functional impairment of mastication [10]. Many of these late complications are best avoided in both orthognathic and trauma surgery. As in orthognathic surgery, similar advantage of CPD was shown in this case, especially during fracture reduction and plating manouvre. In addition, it also justified all three concerns of its usage as previously described for orthognathic surgery, namely, the stability of surgical results, reduced adverse effect to the joints, and improvement of the masticatory functions [12].

Finally, this method represents a simple but significant solution for controlling the proximal segment in an extensively comminuted mandibular fracture. Conventional approach that largely depends on surgeon's experience or even by applying digital pressure to the gonial angle could lead to higher surgical complications. It is therefore for our understanding that this approach could be considered and useful in an extensively comminuted mandible fracture where other options to establish reference during fracture reduction were at no avail.
